# Mannose Receptor Deficiency Impacts Bone Marrow and Circulating Immune Cells during High Fat Diet Induced Obesity

**DOI:** 10.3390/metabo12121205

**Published:** 2022-12-01

**Authors:** Jasmine Nour, Annalisa Moregola, Monika Svecla, Lorenzo Da Dalt, Rossella Bellini, Olivier Neyrolles, Gian Paolo Fadini, Yoann Rombouts, Mattia Albiero, Fabrizia Bonacina, Giuseppe Danilo Norata

**Affiliations:** 1 Department of Excellence of Pharmacological and Biomolecular Sciences, University of Milan, 20133 Milan, Italy; 2 Institut de Pharmacologie et de Biologie Structurale, IPBS, University of Toulouse, CNRS, UPS, 31400 Toulouse, France; 3 Department of Medicine, University of Padua, 35128 Padua, Italy; 4 Veneto Institute of Molecular Medicine, 35129 Padua, Italy; 5 Centro SISA Per lo Studio Dell’Aterosclerosi, Ospedale Bassini, 20092 Cinisello Balsamo, Italy

**Keywords:** Mrc1, obesity, bone marrow, inflammation

## Abstract

The mannose receptor C-type 1 (Mrc1) is a C-type lectin receptor expressed on the immune cells and sinusoidal endothelial cells (ECs) of several tissues, including the bone marrow (BM). Parallel to systemic metabolic alterations and hematopoietic cell proliferation, high-fat diet (HFD) feeding increases the expression of Mrc1 in sinusoidal ECs, thus calling for the investigation of its role in bone marrow cell reprogramming and the metabolic profile during obesity. *Mrc1*^−/−^ mice and wild-type (WT) littermates were fed an HFD (45% Kcal/diet) for 20 weeks. Weight gain was monitored during the diet regimen and glucose and insulin tolerance were assessed. Extensive flow cytometry profiling, histological, and proteomic analyses were performed. After HFD feeding, *Mrc1*^−/−^ mice presented impaired medullary hematopoiesis with reduced myeloid progenitors and mature cells in parallel with an increase in BM adipocytes compared to controls. Accordingly, circulating levels of neutrophils and pro-inflammatory monocytes decreased in *Mrc1*^−/−^ mice together with reduced infiltration of macrophages in the visceral adipose tissue and the liver compared to controls. Liver histological profiling coupled with untargeted proteomic analysis revealed that *Mrc1*^−/−^ mice presented decreased liver steatosis and the downregulation of proteins belonging to pathways involved in liver dysfunction. This profile was reflected by improved glucose and insulin response and reduced weight gain during HFD feeding in *Mrc1*^−/−^ mice compared to controls. Our data show that during HFD feeding, mannose receptor deficiency impacts BM and circulating immune cell subsets, which is associated with reduced systemic inflammation and resistance to obesity development.

## 1. Introduction

The mannose receptor (Mrc1) is a unique type 1 transmembrane receptor that is a member of the C-type lectin family. Mrc1 can be located on non-vascular endothelium, macrophages [1], and dendritic cells [2] as well as in the serum in a shortened soluble form [3]. It presents eight C-type lectin domains (CTLDs), of which only number four is functional, and recognizes mannose, fucose, and N-acetylglucosamine (GlcNac) [4]. Moreover, Mrc1 has a fibronectin type II (FNII) domain with a high affinity for collagen [5], and a terminal cysteine-rich (CR) domain which binds sulfated sugars [6]. 

Because of its complex structure, the mannose receptor recognizes several ligands, including circulating hormones [7,8] and adhesion molecules [9]. This aspect, together with its wide expression, allows Mrc1 to be involved in several processes including the immune response. It has been proposed that Mrc1 plays a key role during the immune response because of its ability to recognize mannose residues [10], expressed as terminal glycan moiety on lysosomal enzymes released during inflammation [11] or as part of microbial walls [12], and because of its participation in leukocyte trafficking. The mannose receptor expressed on sinusoidal endothelial cells interacts with the highly glycosylated protein CD44 on lymphocytes, directly regulating their trafficking [9]. Notably, Mrc1 is also expressed in the bone marrow compartment by both macrophages [13] and sinusoidal endothelium [14], which are known to modulate both the proliferation/maturation of hematopoietic stem cells and the extravasation of immune cells [15]. 

Conditions that cause systemic inflammation, such as metabolic disturbances and obesity [16], impair bone marrow hematopoiesis and promote the production of pro-inflammatory mediators by sinusoidal ECs [17]. The outcomes of these conditions also activate an immune inflammatory response within several target tissues, including the adipose tissue and the liver. The aims of our study were to investigate (1) the role of the mannose receptor in the bone marrow during obesity development and (2) the relationship between the mannose receptor function and changes in systemic inflammation. We fed *Mrc1*^−/−^ and control WT mice a high-fat diet (HFD) for 20 weeks and observed that Mrc1 deficiency resulted in reduced myelopoiesis associated with an increased adipocyte count in the bone marrow. This was associated with a significant reduction of circulating myeloid inflammatory cells together with a reduced infiltration of monocyte-derived macrophages in visceral adipose tissue (VAT) and in the liver. The improved inflammatory response in Mrc1-deficient mice was paralleled by reduced body weight gain during HFD feeding, reduced liver steatosis, and improved glucose and insulin response compared to WT mice. These data suggest that the mannose receptor could be involved in the development of the immunometabolic response associated with diet-induced obesity.

## 2. Materials and Methods

Extensive details of the materials and methods used in our study are provided as an online supplement.

### 2.1. Mice 

*Mrc1*^−/−^ mice on a C57BL/6J background were used and compared to age-matched C57BL/6J wild-type littermates. Four animals were housed per cage and kept in a temperature-controlled environment (20 ± 2°C, 50 ± 5% relative humidity) with a 12 h light/dark cycle and free access to food and water. Six- to 8-week-old male mice were fed a high-fat diet (HFD, 45% Kcal from fat, Cat. No. D12451, Research diets Inc., New Brunswick, NJ, USA) for 20 weeks. Weight gain was measured weakly. All mice were sacrificed after overnight fasting using isoflurane (2%) inhalation, and then blood was collected using an intracardiac puncture. We also collected and weighed visceral adipose tissue (VAT), subcutaneous adipose tissue (SCAT), brown adipose tissue (BAT) and the liver, femurs, and tibias from the mice. All animal procedures conformed to the guidelines from directive 2010/63/EU of the European Parliament on the protection of animals used for scientific purposes and were approved by the Ethical Committee (Progetto di Ricerca 2012/02, Autorizzazione Ministeriale 929/2020).

### 2.2. Glucose and Insulin Tolerance Tests

The glucose tolerance test (GTT) and insulin tolerance test (ITT) were used to measure the plasmatic clearance of glucose after intraperitoneal injection of glucose or insulin, respectively. For the GTT, animals were fasted overnight (approximately 14 h) and then blood glucose levels were measured at fasting and after 20, 40, 60, and 120 min from the injection of a glucose solution (10% *w*/*v* in physiological solution, 1 mg per gram of body weight). Blood glucose levels were measured with a glucometer (ONE-TOUCH Ultra glucometer, Lifescan, Milpitas, CA, USA). For the ITT, animals were fasted for 4 h and then glucose plasma levels were measured at fasting and after 20, 40, 60, and 120 min from the injection of human recombinant insulin (0.75 mU per gram of body weight, Humulin R U-100, 100 U/mL).

### 2.3. Sample Preparation for Flow Cytometry

Immunophenotyping was performed on blood or single-cell suspensions that were previously obtained from bone marrow centrifugation (as described in Fan Y. et al. [18]), visceral adipose tissue collagenase digestion (as previously described [19]), or liver dissociation and stratification. Whole blood was incubated with an antibody mix for 30 min at 4 °C and then fixed and lysed (eBioscience, San Diego, CA, USA) following the manufacturer’s instructions. Cell suspensions from each tissue were stained with the antibody mix at 4 °C for 30 min. Washes and the final resuspension for acquisition were performed using PBS with FBS 2% and EDTA 2 mM. Samples were acquired with LSRFortessa X-20 (BD) and analyzed with Novoexpress software (version 1.6.0, Agilent, Santa Clara, CA, USA). Further details on tissue preparation are provided in the Supplementary Materials. The antibodies used are listed in Supplementary Table S1.

### 2.4. Femurs, Liver, and Adipose Tissues Histology

Mouse femurs were fixed in 4% paraformaldehyde for 4 h at 4 °C, decalcified in 0.5 M EDTA pH 8 for 5 days at 4 °C, and frozen in liquid nitrogen-cooled isopentane. Longitudinal femur sections (10 μm thick) were incubated with anti-perilipin 1 antibody (1:200; Cat. No. 9349, Cell Signalling Technology®, Danvers, MA, USA). An anti-rabbit secondary antibody (Jackson ImmunoResearch, West Grove, PA, USA) diluted to 1:200 in PBS plus 1% BSA was incubated for 40 min at 37 °C. Nuclei were counterstained with Hoechst 33342 (Merck KGaA, Darmstadt, Germany). Images (10×) were acquired with a Leica DM6B automated upright microscope (Leica Biosystems S.r.l., Milan, Italy) and then processed with Fiji/ImageJ software (version 1.50, NIH, Bethesda, MD, USA). Adipocytes were manually quantified with Fiji/ImageJ based on perilipin-1 immunostaining. 

Part of the liver, VAT, SCAT, and BAT was fixed overnight in 4% buffer formalin (Sigma-Aldrich, St. Louis, MO, USA) embedded in paraffin and then 5 µm tissue sections were stained with haematoxylin and eosin (H&E) (Sigma-Aldrich). Images of H&E-stained sections were acquired using the Axiovision Zeiss software (version 4.8, Carl Zeiss, Oberkochen, Germany) at 10× magnification. We evaluated liver steatosis using ImageJ software and the adipocytes area of VAT and SCAT using the Adiposoft Plugin, considering two fields per section, for a total of 10 sections per animal. To assess hepatic neutrophil activation, immunohistochemistry staining of liver sections was performed using the Anti-Neutrophil Elastase antibody (1:200; Cat. No. ab68672, Abcam, Cambridge, UK) followed by the HRP-conjugated anti-rabbit antibody (1:750; Sigma-Aldrich), a reaction with DAB Liquid Substrate System (Sigma-Aldrich), and nuclei counterstaining with haematoxylin (Sigma-Aldrich). Images were acquired with Axiovision Zeiss software at 10x magnification and elastase-positive areas were quantified with ImageJ software.

### 2.5. Proteomics Analysis

A proteomics analysis was performed as previously described [20]. Briefly, mice livers (*n* = 3 WT mice and *n* = 3 *Mrc1*^−/−^) were pooled up to 20 mg and lysed with urea 8M, Tris-HCl 0.1 M pH 8.5 in the presence of protease inhibitors at a ratio of 1:100 (Cell Signalling, Cat. No. 5872S). The liver samples were then quantified and prepared for LC-MS/MS analysis. For plasma, 30 μL samples (*n* = 4 WT mice and *n* = 4 *Mrc1*^−/−^ mice) were pooled, and the concentration was quantified by NanoDrop A280 nm (Thermo Fisher Scientific, Waltham, MA, USA) for LC-MS/MS analysis. Methodological details are provided in the Supplementary Materials.

### 2.6. Quantitative Real-Time PCR (qRT-PCR)

For the gene expression analysis, brown adipose tissue was homogenized with TissueRuptor II (Qiagen, Hilden, Germany), and total RNA was extracted using the RNeasy Lipid Tissue Kit (Qiagen) following the manufacturer’s instructions. RNA was first quantified with a NanoDrop 1000 Spectrophotometer (Thermo Fisher Scientific) and then retro-transcribed to cDNA with iScript™ Reverse Transcription Supermix (Cat. No. 1708841, Bio-Rad, Hercules, CA, USA). A 20 ng sample of the cDNA was used for amplification by real-time quantitative PCR (CFX Connect Real-Time PCR Detection System instrument, Cat. No. 1855201, Bio-Rad) with the Luna^®^ Universal qPCR Master Mix (Cat. No. M3003E, New England Biolabs, Ipswich, MA, USA). For each amplification, 1 µM of each primer was used to a final volume of 15 µL of the reaction mix with 7.5 µL of the master MIX. 

The threshold cycle number (Ct) values for each reaction were calculated. Gene expression was calculated as 2^ΔΔCt^ adjusted to the expression of the housekeeping gene *Rpl-13a* and as a fold difference of *Mrc1*^−/−^ to control group. The primer sequences used in this study are listed in Supplementary Table S2.

### 2.7. Statistical Analysis

GraphPad Prism (version 9, GraphPad Software, San Diego, CA, USA) was used for graphical representation and statistical analyses of the data. Results are given as the mean per group ± standard error of the mean (SEM). For comparison between the two groups, an unpaired parametric two-sided T-test with a 95% confidence interval was used when the data sets followed a normal distribution. For non-normal data, the Mann–Whitney nonparametric unpaired T-test was applied. A *p*-value of <0.05 was considered significant.

The DAVID (The Database for Annotation, Visualization, and Integrated Discovery, NIAID, North Bethesda, MD, USA) platform was used for gene ontology (GO) and KEGG enrichment analyses. GO and KEGG were performed using our protein LFQ dataset, and results for significant terms associated with the gene set were selected based on the FDR < 0.05.

## 3. Results

### 3.1. Mrc1 Deficiency in Mice Fed with a High-Fat Diet Affects Bone Marrow Myelopoiesis and Adiposity, Which Results in Dampened Immune-Inflammatory Response in the Circulation

**Figure 1:**
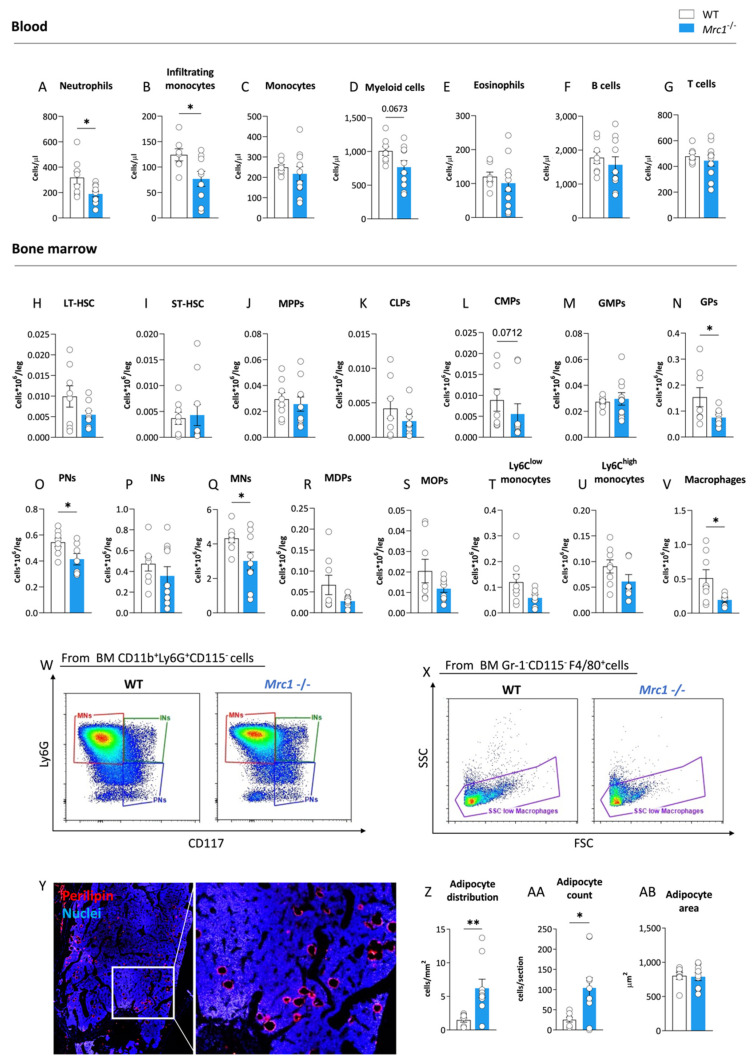
Mrc1 deficiency impact on circulating immune cell subsets and bone marrow hematopoiesis during high-fat diet feeding. Circulating neutrophils (**A**), infiltrating monocytes (**B**), monocytes (**C**), myeloid cells (**D**), eosinophils (**E**), lymphocyte B cells, (**F**) and lymphocyte T cells (**G**) counts in WT and *Mrc1*^−/−^ mice fed an HFD for 20 weeks. Earlier hematopoietic progenitors, including long-term hematopoietic stem cells (LT-HSCs, (**H**)) and short-term hematopoietic stem cells (ST-HSCs, (**I**)), multipotent progenitors (MPPs, (**J**)) and common lymphoid progenitors (CLPs, (**K**)), common myeloid progenitors (CMPs, (**L**)), granulocyte-monocyte progenitors (GMPs, (**M**)), and granulocyte progenitors (GPs, (**N**)). Bone marrow distribution of pre-neutrophils (PNs, (**O**)), immature neutrophils (Ins, (**P**)), mature neutrophils (MNs, (**Q**)), macrophage/dendritic cell progenitors (MDPs, (**R**)), monocyte progenitors (MOPs, (**S**)), Ly6C^low^ monocytes (**T**) and Ly6C^high^ monocytes (**U**), and macrophages (**V**) were profiled in bone marrow from WT and *Mrc1*^−/−^ mice fed with an HFD for 20 weeks. Representative flow cytometry pseudocolor plots (red for higher density of cells, blue for lower) of WT and *Mrc1*-deficient mice of neutrophil differentiation subpopulations and macrophages are shown in panels (**W**,**X**). Representative immunofluorescence acquisition of femur sections stained with perilipin (left) with magnification of the outlined area (right) (**Y**). Adipocyte distribution per area (**Z**), mean count of cells per section (**AA**), and adipocyte area (**AB**) are shown. Results are expressed as mean ± SEM, *n* = 8–10 mice per group. * *p* < 0.05; ** *p* < 0.01.

To investigate whether mannose receptor deficiency affects the immune cell profile during obesity, we first characterized the circulating immune cell profile for the *Mrc1*^−/−^ and control WT male mice fed an HFD for 20 weeks with a focus on myeloid populations (gating strategy shown in Supplementary Figure S1A–F). The *Mrc1*^−/−^ mice displayed a significant reduction in neutrophils (Figure 1A) and inflammatory CCR2^+^ monocyte counts (Figure 1B). Although, the total monocyte numbers were not different between the two experimental groups (Figure 1C). A trend toward the reduction of circulating CD11b^+^ myeloid cells was also observed (Figure 1D), whereas the counts of eosinophils (Figure 1E) and circulating adaptive immune cells, including B and T lymphocytes, were not different (Figure 1F,G). We next investigated whether this profile might be the consequence of changes in the medullary compartment of *Mrc1*^−/−^ as compared to WT male mice following HFD feeding. To this aim, we profiled different hematopoietic precursor subsets and mature immune cells (cell subsets investigated are presented in Supplementary Figure S2, and the gating strategy used is presented in Supplementary Figure S3A–R) in the bone marrow of the different animal models.

The number of early progenitors was similar in WT and *Mrc1*^−/−^ mice, including that of long-term hematopoietic stem cells (LT-HSCs, Figure 1H), short-term hematopoietic stem cells (ST-HSCs, Figure 1I), multipotent progenitors (MPPs, Figure 1J), common lymphoid progenitors (CLPs, Figure 1K), common myeloid progenitors (CMPs, Figure 1L), and granulocyte/macrophage progenitors (GMPs, Figure 1M). Interestingly, the amount of more mature cells, including granulocyte progenitors (GPs, Figure 1N), pre-neutrophils (PNs, Figure 1O), and mature neutrophils (MNs, Figure 1Q, representative plots Figure 1W), were significantly reduced in mice lacking Mrc1 as compared to controls.

Macrophage/dendritic cell progenitors (MDPs; Figure 1R), monocyte precursors (MOPs, Figure 1S), and mature Ly6C^low^ (Figure 1T) and Ly6C^high^ (Figure 1U) monocytes were not affected. On the other hand, macrophages identified as Gr-1^−^CD115^−^F4/80^+^SSC^low^ decreased in the bone marrow of Mrc1-deficient mice compared to WT mice (Figure 1V,X). This last trait was commonly associated with compromised retention of hematopoietic stem cells in the niche [21], often as the consequence of altered crosstalk between the stem cell niche and adipocytes within the bone marrow during hematopoiesis [22,23]. To investigate this hypothesis, we compared the content of adipocytes in the bone marrow of *Mrc1*^−/−^ mice to control mice using Perilipin as an adipocyte marker with immunofluorescence staining (Figure 1Y). Adipocyte content significantly increased in association with Mrc1 deficiency both in terms of cells per area and absolute count (Figure 1Z,AA). The observation of a similar value for adipocyte cell surface area between the experimental groups (Figure 1AB) suggests that an investigation of differences in bone marrow adipogenesis is required to explain the phenotype described above. Altogether, the decreased presence of macrophages, the combination of reduced levels of myeloid precursors and mature cells, and the increased adipocyte content suggest that the lack of Mrc1 could promote a medullary rewiring in mice with diet-induced obesity. This medullary rewiring is reflected in changes to the circulating immune subsets and systemic inflammatory status.

**Figure 2:**
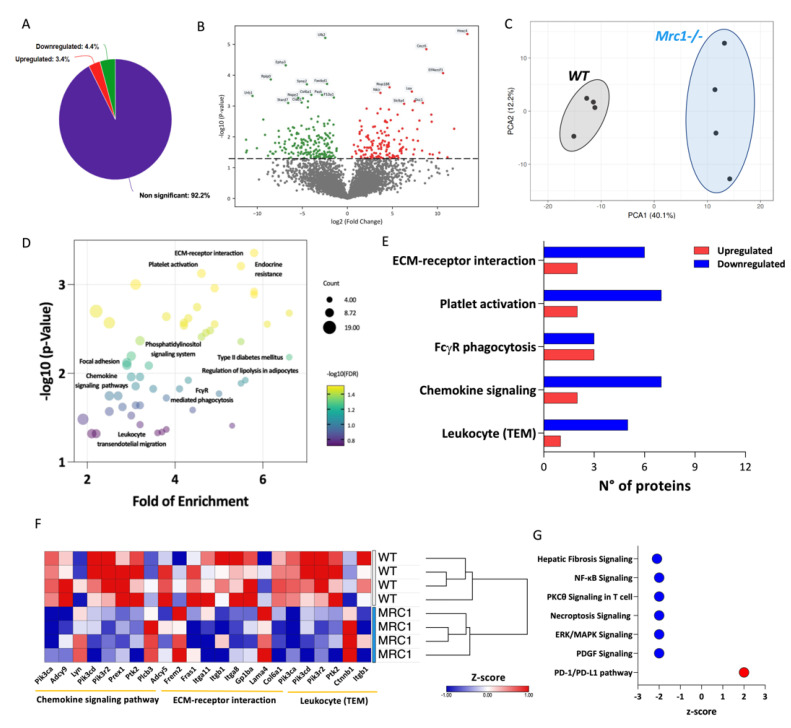
Plasma proteomics profiling of *Mrc1*^−/−^and WT mice fed with an HFD for 20 weeks. (**A**) Percentage of significantly upregulated (red), significantly downregulated (green), and unchanged (purple) proteins in the plasma from *Mrc1*^−/−^mice compared to WT. (**B**) Plasma proteome volcano plot showing log2 fold of change (*x*-axis) and the −log10 *p*-value (*y*-axis) of *Mrc1*^−/−^ mice versus WT (upregulated proteins are shown in red, *p* < 0.05, FC > 1; downregulated proteins are shown in green, *p* < 0.05, FC < −1). (**C**) Principal component analysis (PCA) of the plasma proteome from WT (grey ellipse) and *Mrc1*^−/−^ (blue ellipse). (**D**) Dot plot showing fold of enrichment (*x*-axis) and the –log10 *p*-value (*y*-axis) of enriched pathways following KEGG database analysis. (**E**) The number of up- and downregulated proteins within the enriched pathways following KEGG database analysis. (**F**) Hierarchical clustering and heatmap showing relative protein expression values (Z-score transformed LFQ protein intensities) of significantly different proteins between WT and *Mrc1*^−/−^mice of the indicated KEGG pathways. (**G**) Downregulated or upregulated pathways as per Ingenuity Pathway Analysis (IPA) of plasma proteome profile of *Mrc1*^−/−^ compared to WT mice. Results are expressed as mean ± SEM, *n* = 8–10 mice per group.

To explore this hypothesis, untargeted proteomic analysis of the plasma from the different experimental groups was performed. Out of 4306 proteins identified, 4.4% were downregulated and 3.4% were upregulated in the plasma of obese *Mrc1*^−/−^ mice compared to that of controls (Figure 2A,B). The different proteomic signature associated with Mrc1 deficiency was confirmed by Principal Component Analysis (PCA, Figure 2C), which showed a cluster clearly separated from that of plasma proteins from WT mice. To better characterize this phenotype, differently abundant proteins were analyzed using the KEGG database to assess enriched pathways in the plasma of *Mrc1*^−/−^ mice compared to that of WT mice (Figure 2D). Interestingly, ECM-receptor interaction, leukocyte transendothelial migration, chemokine signaling pathway, and platelet activation (FDR < 0.05) displayed a higher number of downregulated proteins (Figure 2E,F) in the plasma from *Mrc1*^−/−^ mice. These results suggest that the lack of the mannose receptor could affect the modulation of immune cell infiltration.

Furthermore, Ingenuity Pathway Analysis confirmed the impact of Mrc1 deficiency on the modulation of the immune system function. The impact was shown by inhibited canonical pathways (Z-score based) including hepatic fibrosis signaling, NF-kB signaling, PKCθ signaling in T cells, necroptosis, and increased PD-1/PD-L1 pathway (Figure 2G).

Overall, these data suggest that Mrc1 deficiency results in a protective BM phenotype characterized by decreased myelopoiesis and increased presence of quiescence-promoting adipocytes, coupled with changes in plasma proteome associated with a reduced number of pro-inflammatory and tissue-infiltrating leukocytes.

### 3.2. Mrc1 Deficiency in Mice Fed with a High-Fat Diet Impacts Immune Infiltration of Metabolic Tissues during Obesity

**Figure 3:**
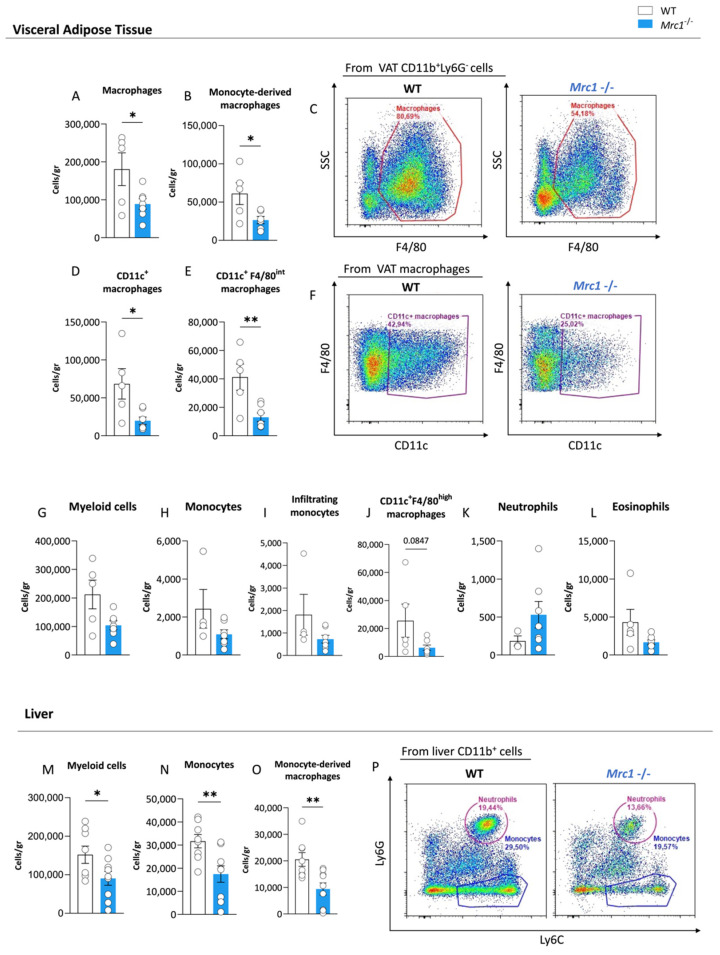
Immune cell subset distribution in visceral adipose tissue and liver from *Mrc1*-deficient and WT mice fed with an HFD for 20 weeks. Macrophages (**A**), CCR2^+^ monocyte-derived macrophages (**B**), and a representative flow cytometry plot of macrophages in WT and *Mrc1*^−/−^ mice (**C**) are shown. Inflammatory CD11c^+^ macrophage count (**D**) and CD11c^+^ F4/80^int^ macrophage count (**E**) in the two experimental groups are shown. A representative flow cytometry plot showing CD11c^+^ macrophages in WT and Mrc1-deficient mice is shown in panel (**F**). VAT counts of myeloid cells (**G**), monocytes (**H**), CCR2^+^ infiltrating monocytes (**I**), CD11c^+^ F4/80^high^ macrophages (**J**), neutrophils (**K**), and eosinophils (**L**) are shown. Liver myeloid cells (**M**), monocytes (**N**) and CCR2^+^ monocyte-derived macrophages (**O**), and representative plots showing neutrophils and monocyte distribution in the two experimental groups are presented in (**P**). Results are expressed as mean ± SEM, *n* = 5–10 mice per group. * *p* < 0.05; ** *p* < 0.01.

We performed flow cytometry analyses on the visceral adipose tissue (VAT) and liver of WT and *Mrc1*^−/−^ mice fed with an HFD for 20 weeks to investigate whether the reduced inflammatory and migratory phenotype of myeloid cells in circulation—as suggested by proteomics analysis—was reflected by different recruitment of immune cells in metabolically affected tissues. Interestingly, the mannose receptor deficiency was associated with a significant reduction in macrophages, specifically in monocyte-derived macrophages (Figure 3A–C, gating strategy shown in Supplementary Figure S4A–I), within VAT, which suggests a different infiltrating capacity. This reduction was paralleled by a significant decrease in inflammatory CD11c^+^ macrophages (Figure 3D,F) in the *Mrc1*^−/−^ mice compared to the controls, which was particularly evident in the subpopulation of macrophages expressing intermediate levels of F4/80 (Figure 3E) that are known to originate from circulating monocytes [24]. A trend toward reduced VAT infiltrating myeloid cells, monocytes, and CD11c^+^F4/80^high^ macrophages was also observed (Figure 3G–J). This trend was not observed for neutrophils and eosinophils (Figure 3K,L).

In parallel, liver immunophenotyping (gating strategy shown in Supplementary Figure S5A–H) showed decreased levels of myeloid cells (Figure 3M) in *Mrc1*^−/−^ mice, which was more evident for monocytes (Figure 3N,P) and monocyte-derived macrophages (Figure 3O) but not for other cell subsets (Supplementary Figure S5I–O). Interestingly, although neutrophils were present in a comparable fashion in both experimental groups (Supplementary Figure S5K), histological analysis of the expression of elastase—a marker of neutrophil activation—on liver sections revealed a trend toward reduction in *Mrc1*^−/−^ mice compared to controls (Supplementary Figure S5P,Q). Such a reduction is in line with a less severe inflammatory phenotype associated with the lack of the mannose receptor.

### 3.3. Immunomodulation in Mrc1^−/−^ Mice Is Associated with Improved Obese Phenotype

**Figure 4:**
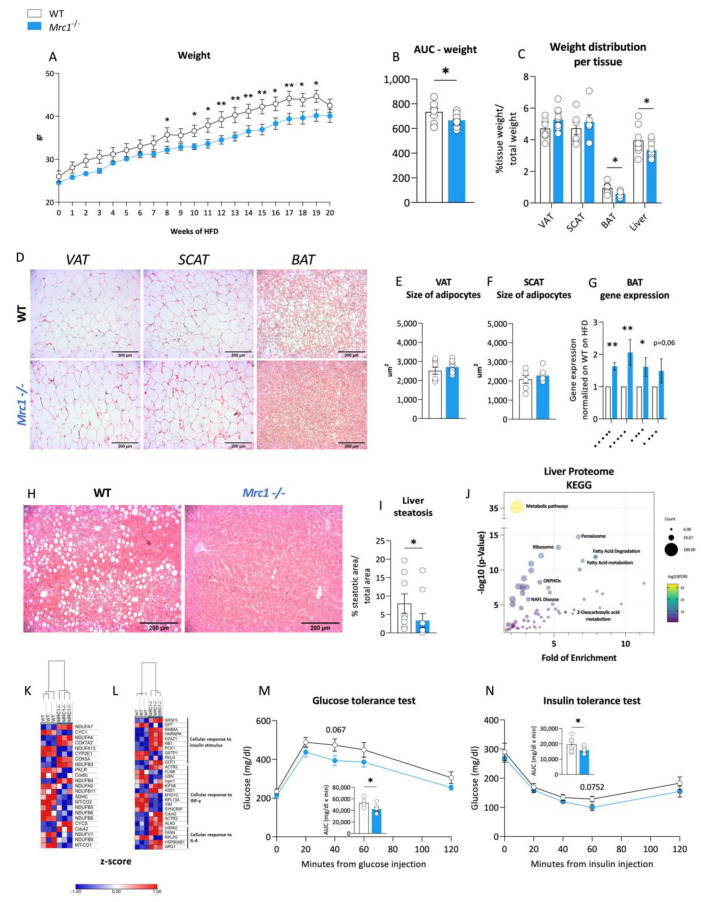
Immune metabolic phenotype of *Mrc1*^−/−^ mice and WT mice fed an HFD for 20 weeks. (**A**) Weekly body weight during 20 weeks of high-fat diet feeding for WT and *Mrc1*^−/−^ mice; (**B**) area under the curve AUC and (**C**) organ/tissue weight distribution of the VAT, SCAT, BAT, and liver from WT and *Mrc1*^−/−^ mice. (**D**) Representative images of hematoxylin and eosin-stained sections from left to right of visceral, subcutaneous, and brown adipose tissues from WT (first row) and *Mrc1*^−/−^ mice (second row). The mean area as um^2^ of adipocytes within the VAT (**E**) and SCAT (**F**) for both experimental groups are presented. (**G**) Gene expression analysis of *Ppara*, *Pgc1a*, *Atgl*, *Ucp1* in BAT from *Mrc1*^−/−^ normalized to that of WT. Representative images of hematoxylin and eosin-stained sections of the liver from both experimental groups (**H**) and the prevalence of liver steatosis (**I**) are shown. (**J**) Dot plot showing fold of enrichment (*x*-axis) and the –log_10_ *p*-value (*y*-axis) of enriched pathways following KEGG database analysis. Hierarchical clustering and heatmap showing relative protein expression values (Z-score transformed LFQ protein intensities) for proteins with significantly different expression in WT and *Mrc1*^−/−^ mice (non-alcoholic fatty liver disease, NAFLD (**K**) and other relevant pathways (**L**),FDR < 0.05). (**M**) Glucose tolerance test (GTT) performed at 20 weeks of HFD feeding in WT and *Mrc1*^−/−^ mice and calculated AUC. (**N**) Insulin tolerance test (ITT) performed at 20 weeks of HFD feeding in WT and *Mrc1*^−/−^ mice and calculated AUC. Blood glucose levels are shown. Glycaemia was measured before i.p. glucose or insulin injection (t = 0, baseline values) and after 20, 40, 60, and 120 min. Results are expressed as mean ± SEM, *n* = 8–10 mice per group. * *p* < 0.05; ** *p* < 0.01.

We compared weight gain in *Mrc1*^−/−^ and WT mice fed a high-fat diet to evaluate whether the observed differences in the immune phenotype translate into an improved metabolic profile. This comparison revealed a significant reduction in the weight gain of *Mrc1*^−/−^ mice compared to their WT counterpart (Figure 4A,B), which was in line with a recent report [25]. This difference was not the result of changes in visceral adipose tissue or subcutaneous adipose tissue. However, significant differences were observed in the relative weight of brown adipose tissue (BAT) and the liver (Figure 4C). Nonetheless, the moderate impact of these differences in explaining the reduction of animal weight suggests that Mrc1 deficiency in mice fed an HFD might affect other tissues that were not evaluated in our work. This limitation should be addressed in future studies. Notably, the adipocyte area was not different in the VAT or SCAT from *Mrc1*^−/−^ and WT mice (Figure 4D–F), but smaller lipid droplets were observed in brown adipose tissue (BAT) (Figure 4D). These smaller lipid droplets were coupled to significant changes in the gene expression profile of genes associated with BAT function such as *Ppara*, *Pgc1a*, *Atgl* and *Ucp1* (Figure 4G).

Reduced lipid accumulation was also observed in the liver (Figure 4H, I) prompting us to profile liver proteome. Among 2114 proteins, 344 were downregulated and 363 were upregulated in *Mrc1*^−/−^ mice (Supplementary Figure S6A,B). Differently abundant proteins were analyzed using the KEGG database to assess enriched pathways (Figure 4J), which were identified as being mainly metabolism related. One of the enriched pathways is non-alcoholic fatty liver disease, or NAFLD, in which most molecules appear less abundant in association with mannose receptor deficiency (Figure 4K), thus confirming the previous histological analysis. On the other hand, the gene ontology (GO) analysis identified the enrichment of the cellular response to the IL-4 pathway, where proteins are mainly upregulated (Figure 4L). This result suggests the presence of pro-resolutive immune activation, which aligns with an overall downregulation of proteins within the cellular response to the IFN-ƴ pathway [26] and with the reduced infiltration observed by flow cytometry. Reduced steatosis and inflammation pair with improved glucose and insulin tolerance, as shown by more upregulated proteins in the GO cellular response to the insulin stimulus pathway (Figure 4L), as well as a better performance following the glucose tolerance test (GTT, Figure 4M) and an improved trend following insulin tolerance test (ITT, Figure 4N). However, no significant changes were observed in plasma triglyceride and cholesterol levels (Supplementary Figure S6C,D). Thus, we observed that in mice fed an HFD for 20 weeks, the absence of the mannose receptor leads to a protective BM phenotype and a reduced leukocyte infiltration tendency, which associates with overall protection from obesity-related dysfunctions.

## 4. Discussion

Metabolic diseases, such as type 2 diabetes mellitus, are associated with increased hematopoiesis [27] and dysfunctional endothelium, which in turn contribute to disrupting myelopoiesis [17]. This pro-inflammatory hematopoiesis not only aggravates the dysmetabolic condition but also stands as an important risk factor for cardiovascular diseases [28], calling for an investigation of the molecular mechanisms driving this effect. Intriguingly, the soluble mannose receptor has been shown to stimulate tissue macrophage activation towards inflammation, and its deficiency in mice fed an HFD resulted in reduced tissue inflammation and a better metabolic phenotype [25].

However, the use of a whole-body *Mrc1* knock-out mouse model has suggested that the wide range in the expression of this receptor [10] would account for additional activities. In this context, we showed that Mrc1 deficiency results in reduced inflammation that might be the consequence of important changes in bone marrow cell signature. 

Interestingly, Mrc1 is expressed by both macrophages and medullary sinusoidal endothelial cells [14], where its expression increases following the onset of diabetes mellitus within a panel of inflammatory genes [17]. Highly active myelopoiesis observed in dysmetabolic conditions is commonly associated with a systemic immune activation characterized by the increased frequency of blood inflammatory cells and circulating markers of inflammation [29]. In our experimental setting, decreased levels of circulating neutrophils and pro-inflammatory CCR2^+^ monocytes were indeed associated with the bone marrow phenotype switch in Mrc1-deficient mice. Our work revealed that the lack of the mannose receptor modulates myelopoiesis, thus hampering the enhancement observed in animals fed with an HFD to induce obesity [30]. We demonstrated an important downregulation of myeloid cell production as well as an increased presence of adipocytes. Considering the role of CXCL12 in promoting HSCs quiescence, an increase in adipocytes, a known source of CXCL12, could negatively modulate hematopoiesis by reducing the production of inflammatory cells [22]. As a confirmation, our plasma proteomics analysis showed that the lack of the mannose receptor is paralleled by an overall reduction in leukocyte migration and the tissue infiltration pathways. Notably, changes in glycoprotein catabolism play a critical role in lipoprotein metabolism and cardiometabolic diseases [31].

Therefore, we investigated the immune phenotype of the visceral adipose tissue and liver, as they represent tissues at the crossroad between the metabolic and inflammatory response to obesity [32,33]. In Mrc1-deficient mice, both tissues presented a reduced infiltration of myeloid cells that contributed to an amelioration of the metabolic response during HFD. Indeed, the recruitment of monocytes from circulation to adipose tissue promotes the differentiation of macrophages with a pro-inflammatory phenotype at the cost of anti-inflammatory macrophages [34].

Notably, Mrc1 deficiency appears to counterbalance this effect, limiting the immunometabolic response observed during obesity. This finding seems unexpected given that the Mrc1 receptor is one of the key markers of pro-resolutive macrophages [35]. However, previous studies have shown that the selective depletion of Mrc1^+^ macrophages improves the systemic insulin response and lipid handling in adipose tissue [36]. These results suggest the presence of parallel mechanisms related to the presence of the mannose receptor that might be associated with the progression of metabolic disorders.

Of note, in this study, we used whole-body *Mrc1* knock-out mice. Therefore, it is possible that cells other than macrophages contributed to the phenotype observed. Indeed, in addition to macrophages, the mannose receptor is expressed by a variety of cells including non-immune cells, such as sinusoidal endothelial cells [1]. These cells have been implicated in leucocyte trafficking in lymphatic vessels [9]. Therefore, future studies should investigate whether leucocyte trafficking could also play a role in the observed phenotype.

In conclusion, we have shown that, during the onset of obesity, Mrc1 impacts hematopoietic stem cell differentiation and release. In addition, Mrc1 deficiency impacts the BM cell profile and reduces the number of activated immune cells in circulation and their infiltration in tissues, thus dampening obesity development.

## Data Availability

The data presented in this study are openly available in FigShare at https://doi.org/10.6084/m9.figshare.21623523.v1 [38].

## Supplementary Materials

The following supporting information can be downloaded at: https://www.mdpi.com/article/10.3390/metabo12121205/s1; Further description of tissue preparation for flow cytometry and proteomics analyses; Figure S1: Blood flow cytometry gating strategy for immunophenotyping of WT and *Mrc1*^−/−^ mice on a high-fat diet; Figure S2: Hematopoiesis diagram; Figure S3: Gating strategy for bone marrow immunophenotyping [37]; Figure S4: Visceral adipose tissue flow cytometry gating strategy for immunophenotyping of WT and *Mrc1*^−/−^ mice with diet-induced obesity; Figure S5: Liver flow cytometry gating strategy and liver immune characterization of WT and *Mrc1*^−/−^ mice fed with HFD; Figure S6: Impact of Mrc1 deficiency on the metabolic phenotype in mice with diet-induced obesity; Table S1: Antibodies used for flow cytometry analyses; Table S2: Primer sequences used for real time qPCR on BAT samples.
