# Supplementary material for: Mannose Receptor Deficiency Impacts Bone Marrow and Circulating Immune Cells during High Fat Diet Induced Obesity
Source: Metabolites. 2022 Dec 1;12(12):1205. doi: 10.3390/metabo12121205 (PMC9784906; doi:10.3390/metabo12121205)
Supplement: Supplementary file 1 [file metabolites-12-01205-s001.zip › supplementary revised.pdf]

SUPPLEMENTARY MATERIALS

Tissue preparation for flow cytometry

One femur and one tibia per mouse were used to perform BM immunophenotyping. Both bones were removed of their extremities and placed in 0,6 ml tubes with a cut open bottom, placed in a 1.5 ml tube. Samples were briefly spun for 10 seconds at 16000 rcf to recover bone marrow and red blood cells were lysed with RBC lysis buffer (eBioscience). Cells were then counted, and $3*10^6$ cells were used for flow cytometry staining.

Visceral adipose tissue was placed in a six-well plate on ice and cut in small pieces in 2mL of PBS 5% BSA solution. For digestion collagenase (2mg/mL final concentration, NB4 standard grade, SERVA Electrophoresis GmbH, Heidelberg, Germany) and $CaCl_2$ (5mM final concentration) were added, and samples were incubated at 37°C for 30 minutes under agitation. Lysis was stopped with PBS, 2% FBS, 2mM EDTA and filtered on a sterile bandage and subsequently on a 100 µm and 70 µm cells strainer. After centrifugation 5 minutes at 1800 rpm, red blood cells were lysed with RBC lysis buffer for 5 minutes on ice, then samples were washed, centrifuged 5 minutes at 1800 rpm and used whole for flow cytometry staining.

Liver was passed through 100 µm cell-strainers with PBS with FBS 2% and EDTA 2mM to disrupt and centrifuged at 50 rcf for brake off for 1 minute to pellet debris. Supernatant was recovered and centrifuged at 700g high-brake for 8 minutes at RT. The pellet was then resuspended in 10 ml of 37.5% Percoll (Sigma-Aldrich), 3.75% di PBS 10x, 58.75% di RPMI 1640 (Euroclone S.p.A., Milan, Italy) and spun at 850g brake off for 30 minutes at RT. The remaining debris stratified in the upper layer was discarded and the recovered pellet was incubated in RBC lysis buffer for 5 minutes on ice, washed and used for flow cytometry staining.

Proteomics

Sample preparation

Proteomics analysis was performed as previously described (Svecla M., et al. Proteomics 2021 [21]). Briefly, mice liver (n=3 WT mice and n=3 *Mrc1*$^{-/-}$) were pooled up to 20 mg and lysed with urea 8M, Tris-HCl 0.1 M pH 8.5 in the presence of protease inhibitors at a ratio of 1:100 (Cell Signaling, Cat# 5872S) for 60 minutes at 4°C with constant shaking. Next, samples were centrifuged for 30 minutes at 14000 g at 4°C. The supernatant containing the proteins extracted was collected and quantified by Lowry protein assay. For the plasma 30 µl (n=4 WT mice and n=4 *Mrc1*$^{-/-}$) were pooled and the concentration was quantified by NanoDrop A280nm (Thermo Fisher). Liver protein extracts were then dried completely using a vacuum concentrator at 45°C for 45 minutes and later resuspended in 10 µl of water with the addition of 10 µl of Ammonium bicarbonate solution 50 mM. (Final pH 8.5). The concentration of 1 µg/µl for plasma was used for additional 30 µl of Ammonium bicarbonate solution 50 mM (final pH 8.5). Proteins were reduced following incubation with DTT (final concentration 5 mM for liver, 6mM for plasma), for 30 minutes at 55°C. Protein alkylation was then performed at RT, by incubating with iodoacetamide (final concentration 15 mM), for 30 minutes in the dark. Trypsin digestion (enzyme to protein ratio of 1:20), was performed overnight at 37°C, and stopped by acidification trifluoroacetic acid, (final percentage: 1%).

LC-MS/MS Analysis

Samples were analyzed using a Dionex Ultimate 3000 nano-LC system (Sunnyvale CA, US) connected to an orbitrap Fusion™ Tribrid™ Mass Spectrometer (Thermo Scientific, Bremen, Germany) equipped with a nanoelectrospray ion source operating in positive ion mode. The peptide mixtures were pre-concentrated onto an Acclaim PepMap C18 5 µm, 100 Å, 100 µm ID x 2cm (Thermo Scientific) and separated at 35°C on an EASY-Spray PepMap RSLC C18 column: 3 µm, 100 Å, 75 µm ID x 25 cm (Thermo Scientific), using mobile phase A (0.1% aqueous formic acid) and mobile phase B (0.1% aqueous formic acid /acetonitrile (2:8)) at a flow rate: 300 nL/min. MS spectra were collected over an m/z range of 375 – 1500 Da at resolution 120.000 in the data dependent mode, cycle time 3 sec between master scans. Fragmentation was induced by higher energy

collisional dissociation (HCD) with collision energy set at 35 eV. Liver samples were analysed in triplicate while plasma samples in quadruplicates.

Data processing and analysis

MS raw data files were converted to mzML format (centroid mode) using the MSconvert tool of the software ProteoWizard (version 3.0.1957, Palo Alto,CA, US). MzML files were then analyzed using an OpenMS (version 2.4, deNBI, Germany) nodes operating within the open-source software platform KNIME® (version 4.1.1, Knime AG, Zurich, Switzerland). Peptides identification was done using a peptides identification approach combining the search engines X!Tandem, MS-GF+ (Sangtae and Pevzner), Novor and SpectraST, against a mouse Uniprot FASTA database (uniprot-mus+musculus.fasta, downloaded at www.uniprot.org), and a common contaminant proteins database. The spectral library required by the SpectraST search engine was downloaded from the website www.peptideatlas.org (file NIST_mouse_IT_2012-04-21_7AA.splib). Peptide sequences were indexed through the OpenMS PeptideIndexer node, setting leucine/isoleucine equivalence. Protein inference was then carried out using the Protein Inference Analysis (PIA) algorithm using the default parameters set by the developers. Protein abundance estimates were calculated with prior generation of spectral feature by the node FeatureFinderMultiplex followed by PIA-assisted FDR-multiple scores estimation and filtering (combined FDR score<0.01), their ID mapping and combination with peptide IDs, their subsequent alignment, grouping and normalization (e.g., MapAlignerIdentification, FeatureUnlabeledQT and ConsensusmapNormalizer nodes). Proteins and peptides label free quantification (LFQ) was then computed with the OpenMS ProteinQuantifier node based on intensities of the n=3 most abundant identified peptides. The corresponding output files were read as tables of the CSVreader node output and exported into Microsoft Office Excel 2016 (Microsoft, Redmon, Washington) for further formatting and statistical elaboration.

Table S1 Antibodies used for flow cytometry analyses

**BM early progenitors**	
Anti-mouse Lineage PerCP-Cy5.5	BD bioscience
Anti-mouse Il-7ra/CD127 PE	Biolegend
Anti-mouse SCA BV421	BD bioscience
Anti-mouse CD117 PE-Cy7	BD bioscience
Anti-mouse CD34 FITC	BD bioscience
Anti-mouse CD16/32 BV650	BD bioscience
Anti-mouse CD48 APC-Cy7	Biolegend
Anti-mouse CD150 BV650	Biolegend
**BM macrophages and advanced progenitors**	
Anti-mouse Ly6C FITC	BD bioscience
Anti-mouse Ly6G PE-eFluor610	Invitrogen
Anti-mouse CD115 BV510	BD bioscience
Anti-mouse F4/80 APC-Cy7	Biolegend
Anti-mouse Lineage PerCP-Cy5.5	BD bioscience
Anti-mouse CD117 PE-Cy7	BD bioscience
Anti-mouse CD16/32 BV650	BD bioscience
Anti-mouse CD11b Af700	Biolegend
**Blood**	
Anti-mouse CD45 BUV563	BD bioscience
Anti-mouse CD3 PerCP-Cy5.5	BD bioscience
Anti-mouse CD19 PE-Cy7	Biolegend
Anti-mouse CD11b BUV737	BD bioscience
Anti-mouse Ly6G FITC	BD bioscience
Anti-mouse Ly6C eF450	Invitrogen
Anti-mouse CCR2 BV650	BD bioscience
**VAT**	
Anti-mouse CD45 BUV563	BD bioscience
Anti-mouse CD11b BUV737	BD bioscience
Anti-mouse Ly6G FITC	BD bioscience
Anti-mouse Ly6C eF450	Invitrogen
Anti-mouse CCR2 BV650	BD bioscience
Anti-mouse F4/80 APC-Cy7	Biolegend
Anti-mouse CD11c BV786	BD bioscience
**Liver**	
Anti-mouse CD45 BUV563	BD bioscience
Anti-mouse CD3 PerCP-Cy5.5	BD bioscience
Anti-mouse CD19 PE-Cy7	Biolegend
Anti-mouse CD11b BUV737	BD bioscience
Anti-mouse Ly6G FITC	BD bioscience
Anti-mouse Ly6C eF450	Invitrogen
Anti-mouse CCR2 BV650	BD bioscience
Anti-mouse F4/80 APC-Cy7	Biolegend
Anti-mouse CD11c BV786	BD bioscience

Table S2 Primer sequences used for real time qPCR on BAT samples

**Gene**	**Fw 5' → 3'**	**Rev 5' → 3'**
Rpl-13a	GCGCCTCAAGGTGTTGGAT	GAGCAGCAGGGACCACCAT
Ppara	CCTCAGGGTACCACTACGGAGTT	CCGAATAGTTCGCCGAAAGA
Pgc1a	TGCCTTCATGCTGTGGTAAGTACT	AAAACCCCGCATTTCTAAAGC
Atgl	TGCAAACAGGGCTACAGAGA	AGCAGGGCATTCCTCCTAAG
Ucp1	ACACTTTGGAAAGGGACGAC	TAAATGGCAGGGGACGTCAT

Supplementary figures legends

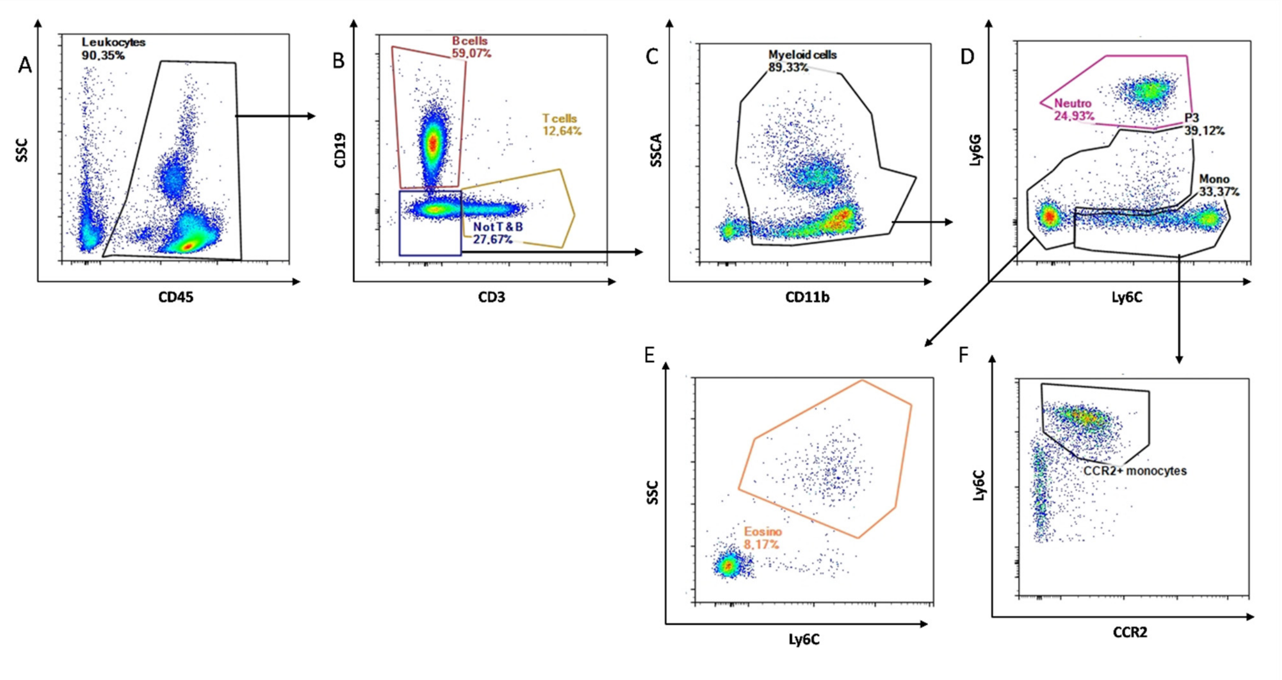

Supplementary Figure S1 – Blood flow cytometry gating strategy for immunophenotyping of WT and *Mrc1*$^{-/-}$ mice on high fat diet.

Gating strategy used for flow cytometry analysis for the indicated cell populations in blood. First, leukocytes were identified as CD45^{+} cells (A), then lymphocyte B cells and lymphocyte T cells were identified based on the expression of CD19 and CD3 respectively (B). From CD19^{-} CD3^{-} cells, myeloid cells were identified based on their expression of CD11b (C). Within myeloid cells, Ly6G expression was used to identify neutrophils while Ly6C to identify monocytes, whereas eosinophils were identified from the Ly6G/Ly6C$^{low\text{-}neg}$ population (P3) as SSC^{high} (E). Inflammatory monocytes were gated as CCR2^{+} Ly6C^{+} cells (F).

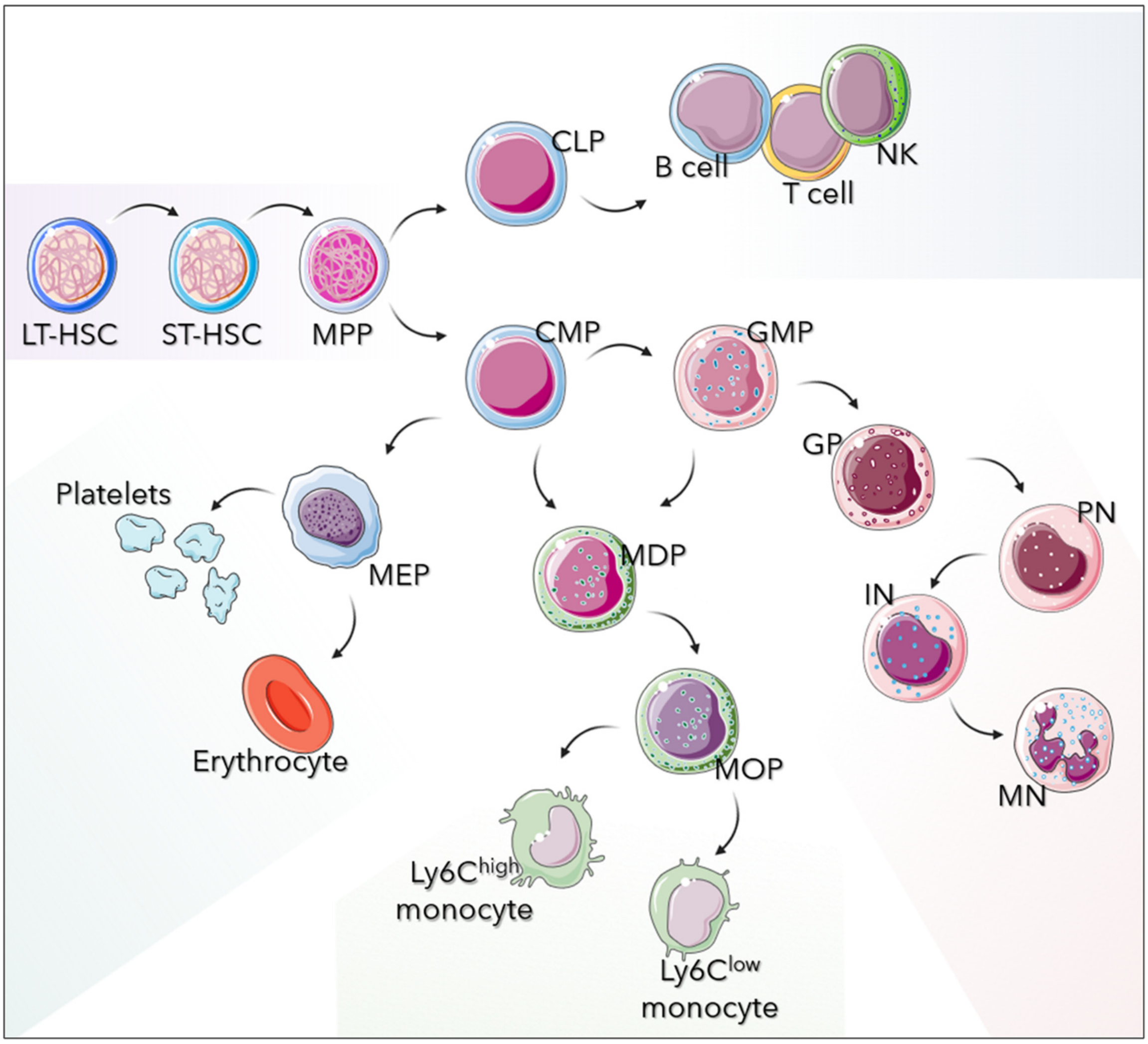

Supplementary Figure S2. Hematopoiesis diagram

Representative diagram showing hematopoietic flux according from long-term hematopoietic stem cells to different mature populations. LT-HSC, long-term hematopoietic stem cell; ST-HSC, short-term hematopoietic stem cell; MPP, multipotent progenitor; CLP, common lymphoid progenitor; NK, natural killer cell; CMP, common myeloid progenitor; MEP, megakaryocyte progenitor; MDP, macrophage/dendritic progenitor; MOP, monocyte progenitor; GMP, granulocyte/macrophage progenitor; GP, granulocyte progenitor; PN, pre-neutrophil; IN, immature neutrophil; MN, mature neutrophil were determined in the bone marrow of the experimental groups.

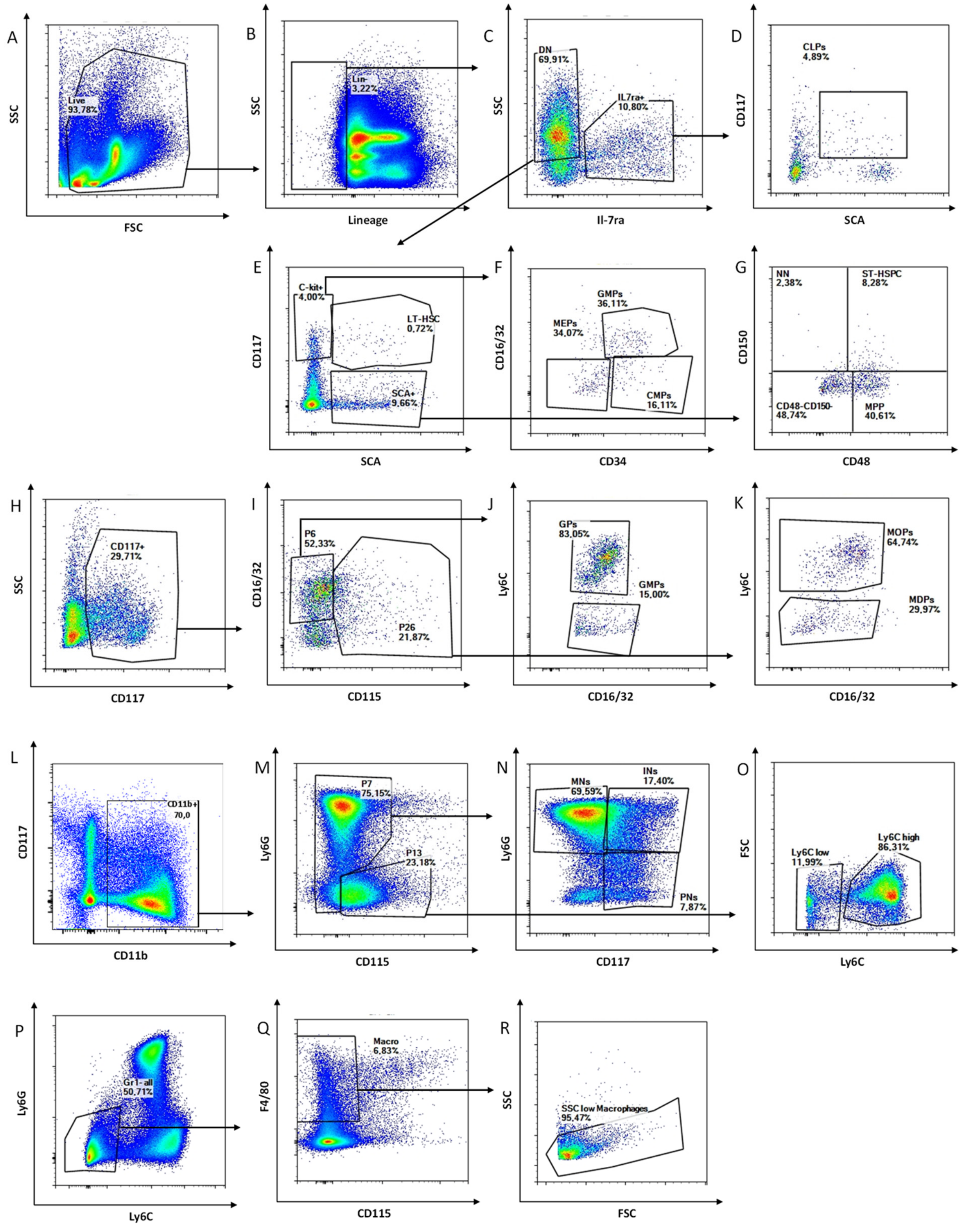

Supplementary Figure S3. Gating strategy for bone marrow immunophenotyping

Gating strategy used for flow cytometry analysis of the cell subsets within the bone marrow. For all characterizations cells were pre-gated on singlets and identified with FSC and SSC (panel A).

To identify early progenitors, the gating strategy previously presented by Schoedel K.B., *et al*. [37] was used. On Lin$^-$ cells (B) Il-7ra$^+$ cells and double negative (DN) were gated as shown (C). From Il-7ra$^+$ cells, common lymphoid progenitors (CLPs) were identified as CD117$^+$SCA$^+$ (D), while from DN cells c-kit$^+$ cells (CD117$^+$SCA$^-$), long-term hematopoietic stem cells (CD117$^+$SCA$^+$) and SCA$^+$ (CD117$^-$SCA$^+$) cells were gated (E). Within c-kit$^+$

cells, granulocyte monocyte progenitors (GMPs) were characterized as CD16/32$^+$CD34$^+$, common myeloid progenitors (CMPs) as CD16/32$^-$CD34$^+$ and megakaryocyte progenitors (MEPs) as CD16/32$^-$CD34$^-$ (F). From SCA$^+$ cells, multipotent progenitors (MPPs) were classified as CD48$^+$ and short-term hematopoietic stem cells (ST-HSCs) as CD48$^+$CD150$^+$(G).

To identify myelopoietic progenitors, from Lin$^-$ cells CD117$^+$ population was identified (H). We further assessed expression of CD16/32 and CD115 (I), and from CD16/32$^+$CD115$^-$ cells we classified granulocyte progenitors (GPs) and granulocyte monocyte progenitors (GMPs) as CD16/32$^+$Ly6c^{high} and CD16/32$^+$Ly6C^{low} respectively (J). In the same way, from CD115$^+$ cells monocyte progenitors (MOPs) and macrophage/dendritic cell progenitor (MDPs) were identified based on high and low expression of Ly6C (K). To characterize differentiated myeloid cells, from CD11b+ cells on total cells (L) subpopulations P7 and P13 based on Ly6G and CD115 expression were identified (M). Within P7 we identified pre-neutrophils (PNs) as CD117$^+$Ly6G$^-$, intermediate neutrophils as CD117$^+$Ly6G$^+$ and mature neutrophils (MNs) as CD117$^-$Ly6G$^+$ (N). Among P13 CD115$^+$ cells we identified Ly6C^{low} and Ly6C^{high} monocytes (O). To identify macrophages, from Ly6C$^-$ and Ly6G$^-$ cells (Gr-1$^-$; P), macrophages were first identified as CD115$^-$ and F4/80$^+$ (Q) and further confirmed as SSC^{low} as shown in panel (R).

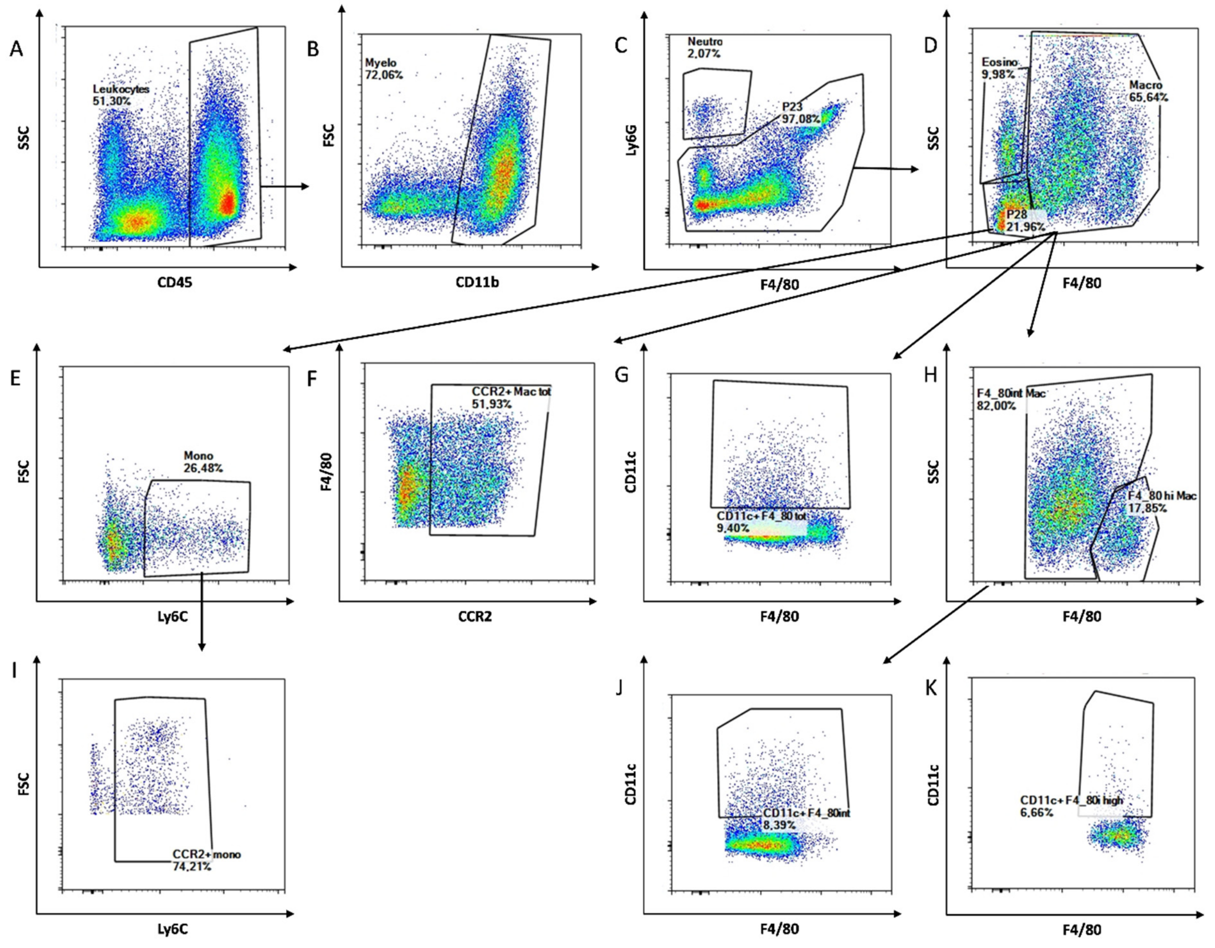

Supplementary Figure S4 – Visceral adipose tissue flow cytometry gating strategy for immunophenotyping of WT and *Mrc1$^{-/-}$* mice with diet-induced obesity.

Gating strategy used for flow cytometry analyses for the indicated cell populations in visceral adipose tissue. First, leukocytes were identified as CD45$^+$ cells (A) then myeloid cells were identified based on their expression of CD11b (B). From this population, Ly6G and F4/80 expressions were used to identify neutrophils as Ly6G$^+$F4/80$^-$ (C); while from other myeloid cells macrophages were identified as F4/80$^+$ and eosinophils as

F4/80$^-$SSC^{high} (D). Monocytes were gated from SSC^{low} and F4/80$^-$ based on their expression of Ly6C (E) and infiltrating monocytes were identified as CCR2$^+$Ly6C$^+$(I). From total macrophages we characterized monocyte-derived cells as CCR2$^+$ (F) and inflammatory macrophages as CD11c$^+$ (G). Macrophages subpopulations were also clustered based on their expression of F4/80, as macrophages derived from monocytes express intermediate levels of F4/80 while resident macrophages express high levels of this marker (H). Expression of inflammatory marker CD11c$^+$ was evaluated in both populations (respectively J and K).

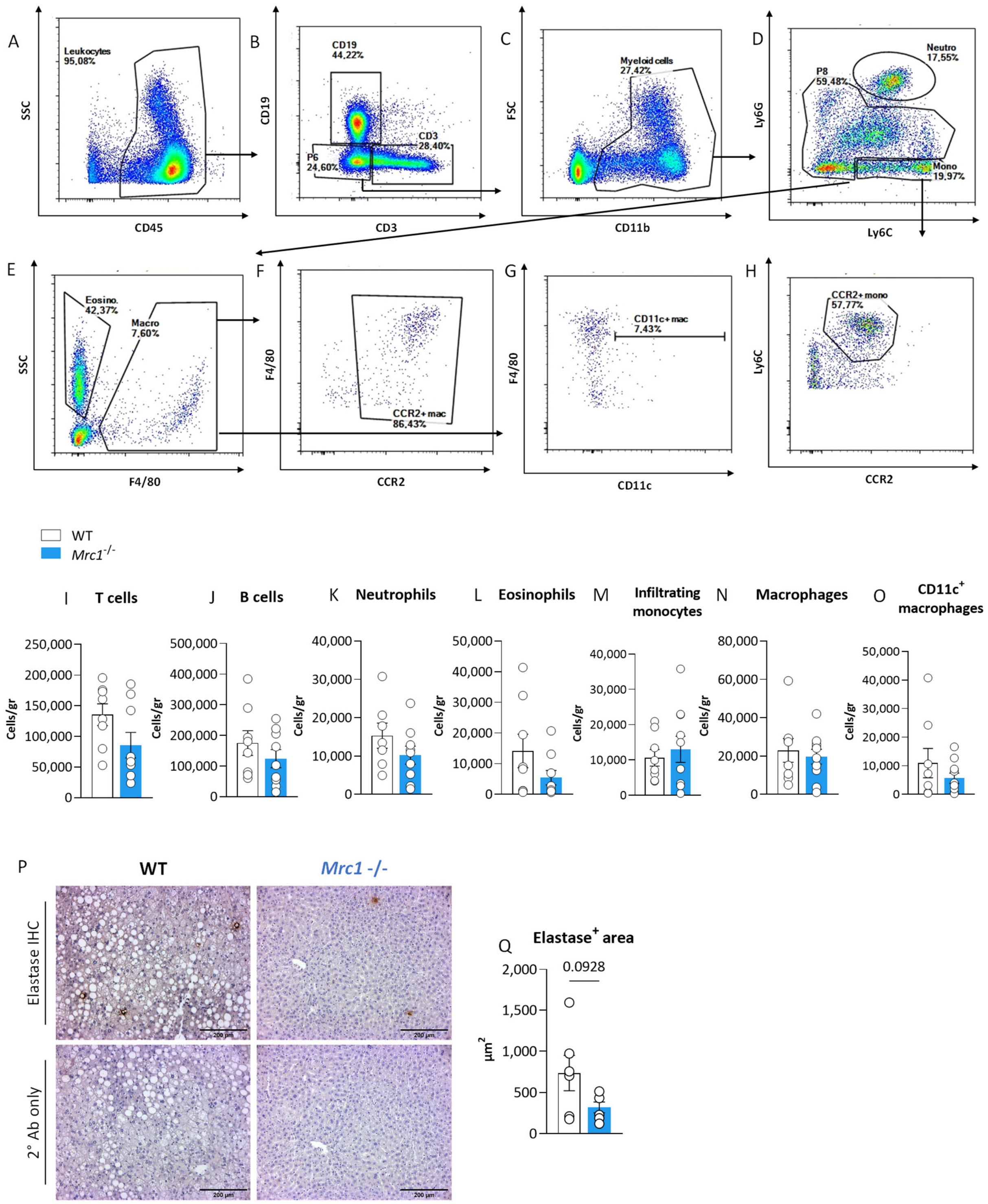

Supplementary Figure S5 – Liver flow cytometry gating strategy and liver immune characterization of WT and *Mrc1*$^{-/-}$ mice fed with HFD.

Gating strategy used for flow cytometry analyses for the indicated cell populations in liver. First, leukocytes were identified as CD45$^+$ cells (A) then lymphocyte B cells and lymphocyte T cells were identified as CD19$^+$ and CD3$^+$ respectively (B). From CD19$^-$CD3$^-$ cells, myeloid cells were identified as CD11b$^+$ (C). Neutrophils were gated as Ly6G$^+$ and monocytes as Ly6C$^+$ (D), while within remaining myeloid cells eosinophils and macrophages were gated as SSC^{high}F4/80$^-$ and F4/80$^+$ respectively (E). CCR2$^+$ monocyte-derived macrophages were gated as shown from total macrophages (F) and inflammatory macrophages as CD11c$^+$ (G). From monocytes, inflammatory CCR2$^+$ cells were identified as in panel (H).

Panels reporting liver counts of lymphocyte T cells (I), lymphocyte B cells (J), neutrophils (K), eosinophils (L), infiltrating monocytes (M), macrophages (N), and CD11c$^+$ macrophages (O) in WT and *Mrc1*$^{-/-}$ mice with diet-induced obesity are shown.

Representative 10x images of immunohistochemistry staining of WT and *Mrc1*$^{-/-}$ liver sections with anti-elastase antibody to mark neutrophil activation (P – upper row) and control staining with secondary antibody only (P – lower row), and panel reporting quantification of mean elastase$^+$ area in both groups (Q). Results are expressed as mean ±SEM, n=8-10 mice per group.

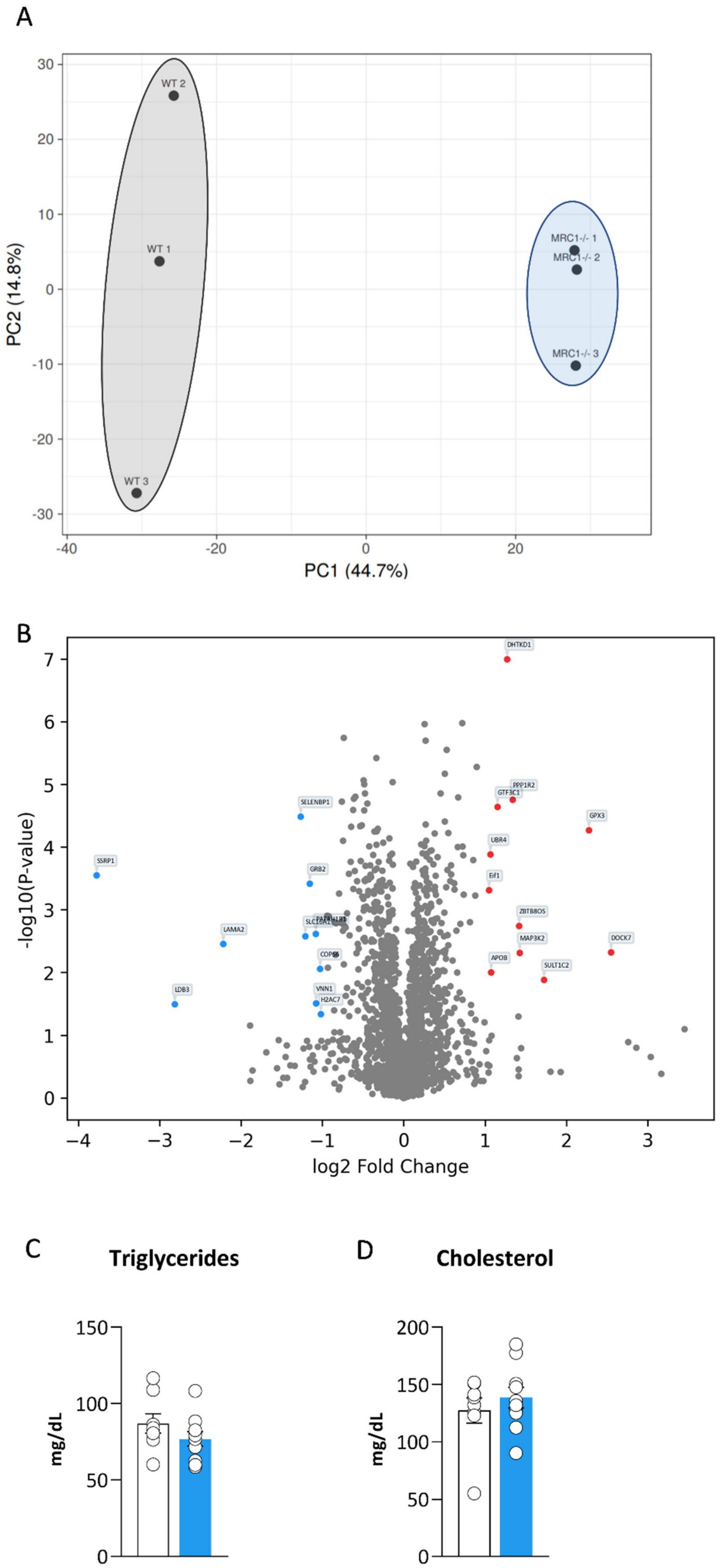
A
PC1 (44.7%)
PC2 (14.8%)
WT 1
WT 2
WT 3
MRC1-/- 1
MRC1-/- 2
MRC1-/- 3
B
log2 Fold Change
-log10(P-value)
DHTKD1
PPP1R2
GTF3C1
GPX3
SELENBP1
UBR4
SSRP1
GRB2
Eif1
ZBTB8OS
LAMA2
MAP3K2
DOCK7
APOB
SULT1C2
VNN1
H2AC7
LDB3
C
Triglycerides
mg/dL
D
Cholesterol
mg/dL

Supplementary Figure S6 – Impact of *Mrc1* deficiency on the metabolic phenotype in mice with diet-induced obesity.

Principal component analysis (PCA) of the liver proteome from WT (grey ellipse) and *Mrc1*$^{-/-}$ (blue ellipse) (A). Liver proteome volcano plot showing $\log_2$fold of change (x-axis) and the $-\log_{10}$ P-value (y-axis) of WT versus *Mrc1*$^{-/-}$ mice (B). Main increased proteins are marked in red ($p < 0.05$, FC> 1), while decreased in blue ($p < 0.05$, FC< -1). Results are expressed as mean ±SEM, n=8-10 mice per group, n=3 technical replicates for liver proteomics analysis. Plasma triglycerides (D) and cholesterol after during 20 weeks of high fat diet for WT and *Mrc1*$^{-/-}$ animals are shown.
