# Supplementary figures and images for: Mannose Receptor Deficiency Impacts Bone Marrow and Circulating Immune Cells during High Fat Diet Induced Obesity
Source: Metabolites. 2022 Dec 1;12(12):1205. doi: 10.3390/metabo12121205 (PMC9784906; doi:10.3390/metabo12121205)

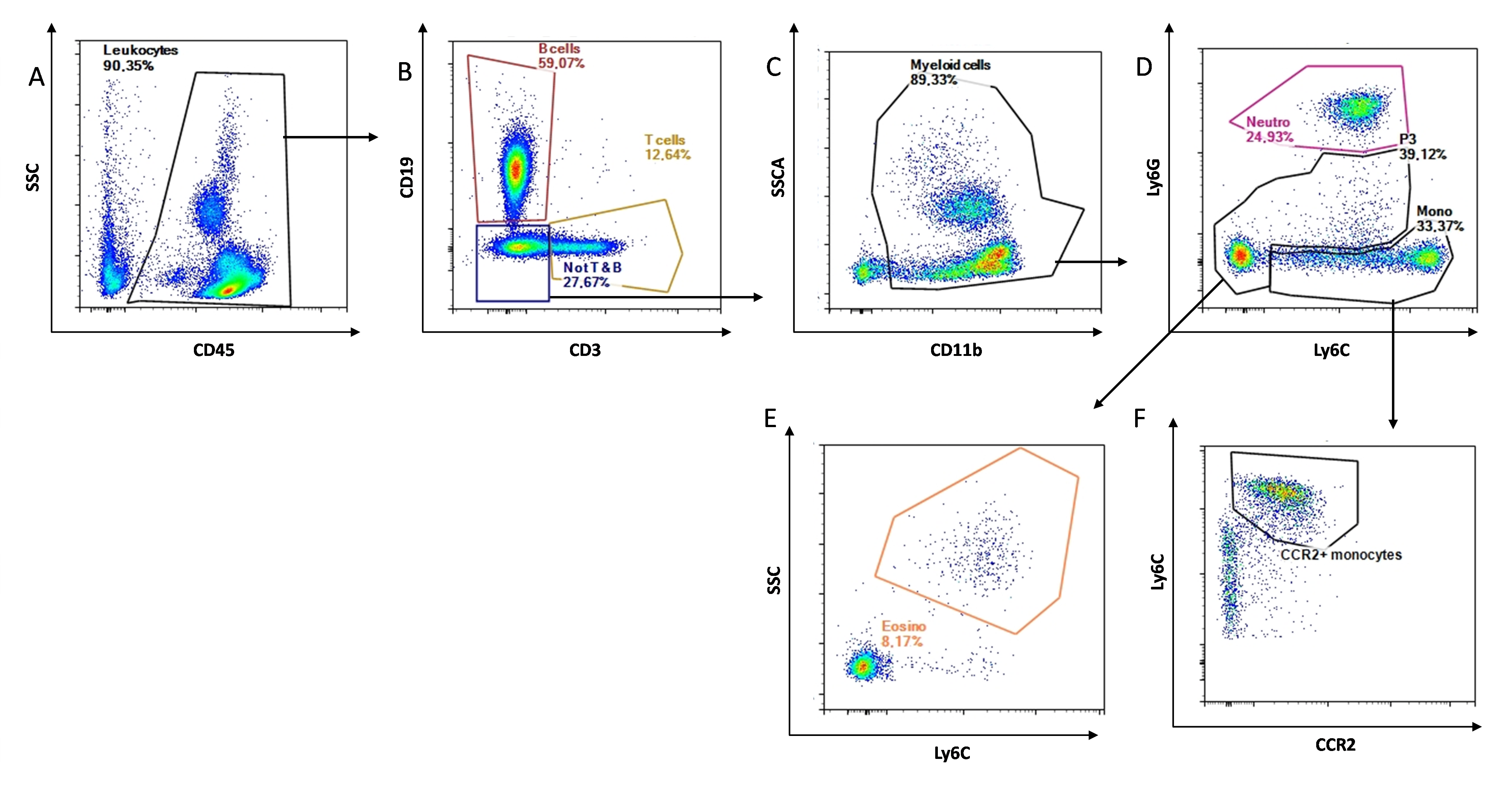

Supplement: Supplementary file 1 [file metabolites-12-01205-s001.zip › Supplementary Figure 1.png]

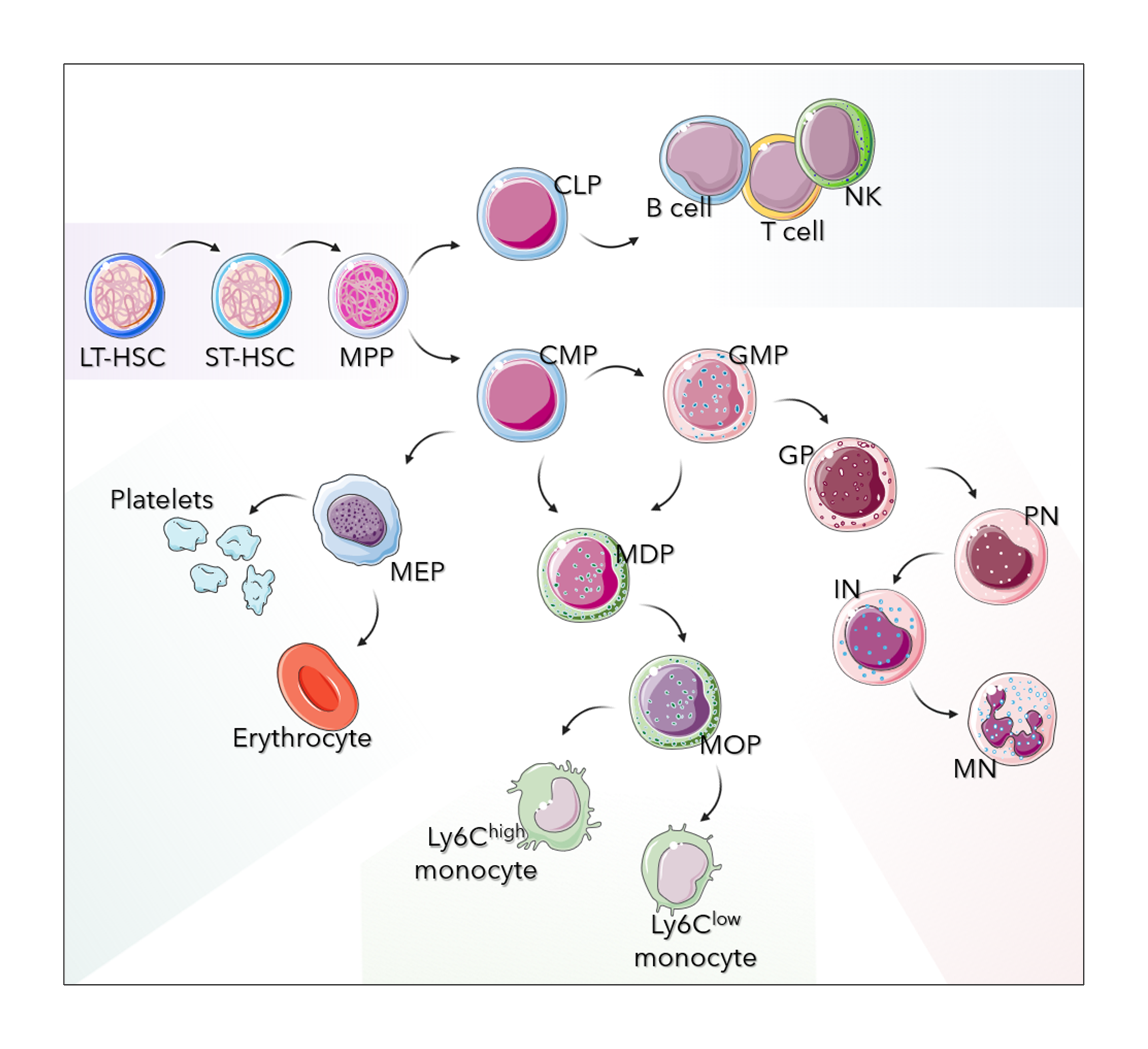

Supplement: Supplementary file 1 [file metabolites-12-01205-s001.zip › Supplementary Figure 2.png]

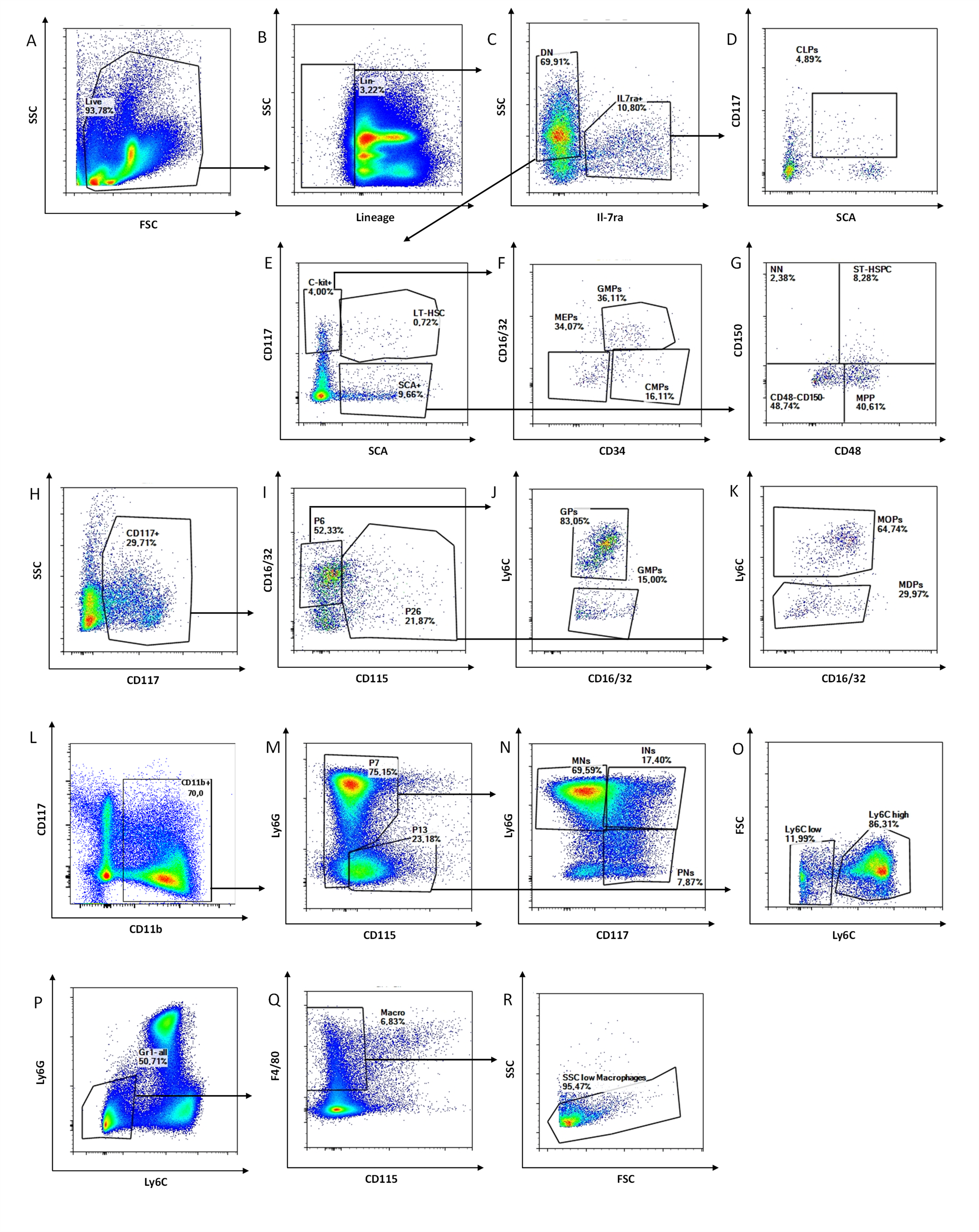

Supplement: Supplementary file 1 [file metabolites-12-01205-s001.zip › Supplementary Figure 3.png]

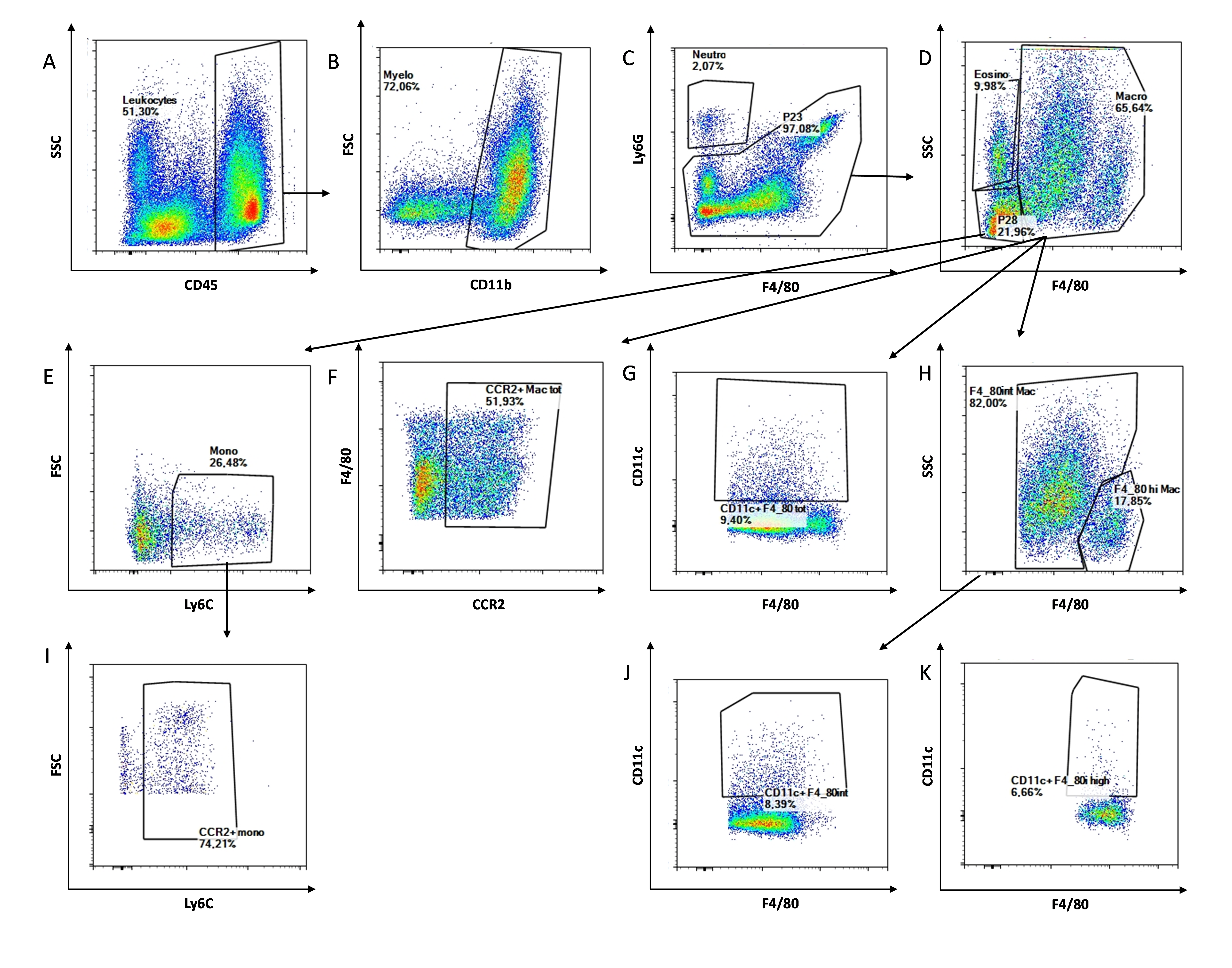

Supplement: Supplementary file 1 [file metabolites-12-01205-s001.zip › Supplementary Figure 4.png]

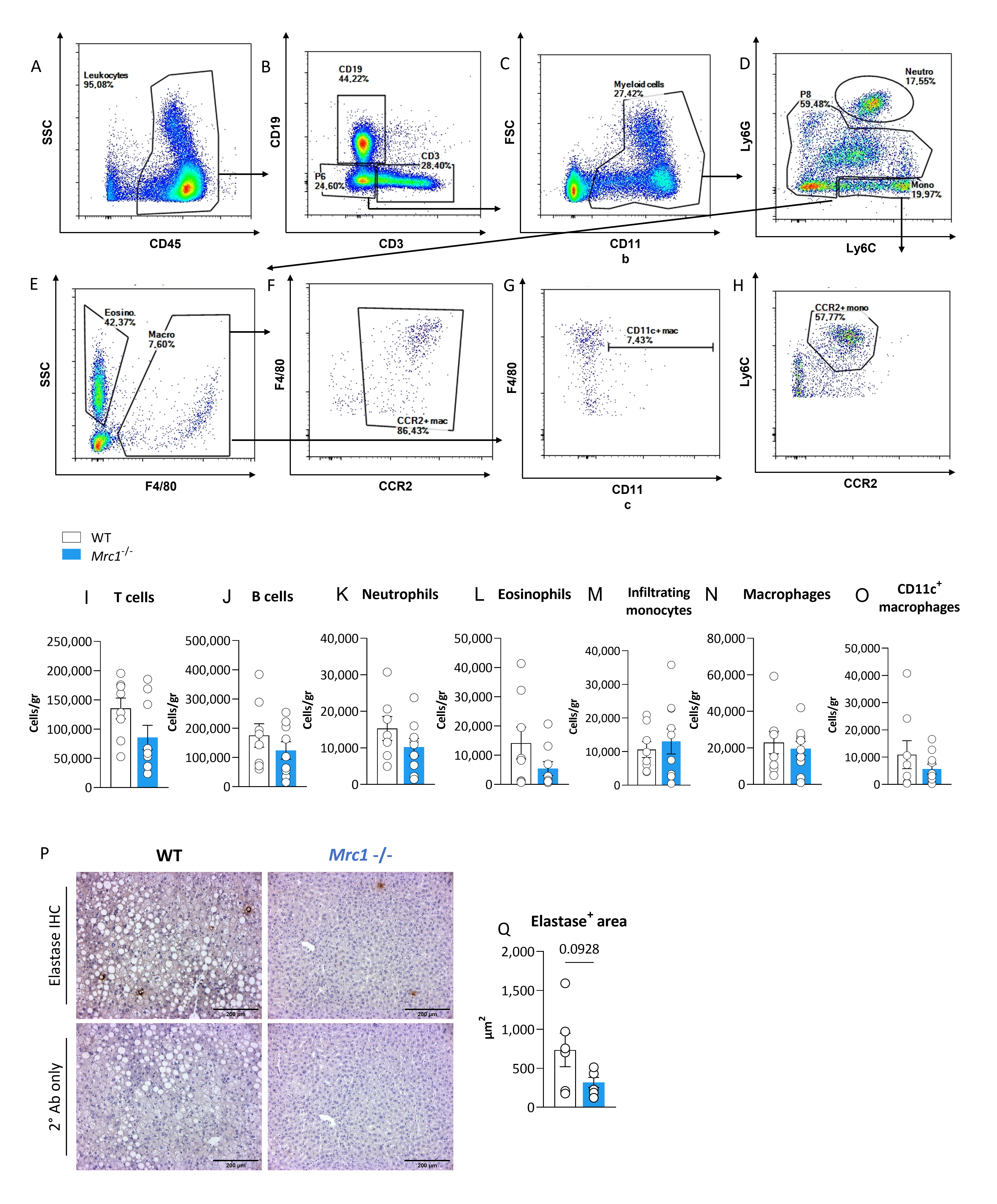

Supplement: Supplementary file 1 [file metabolites-12-01205-s001.zip › Supplementary Figure 5.png]

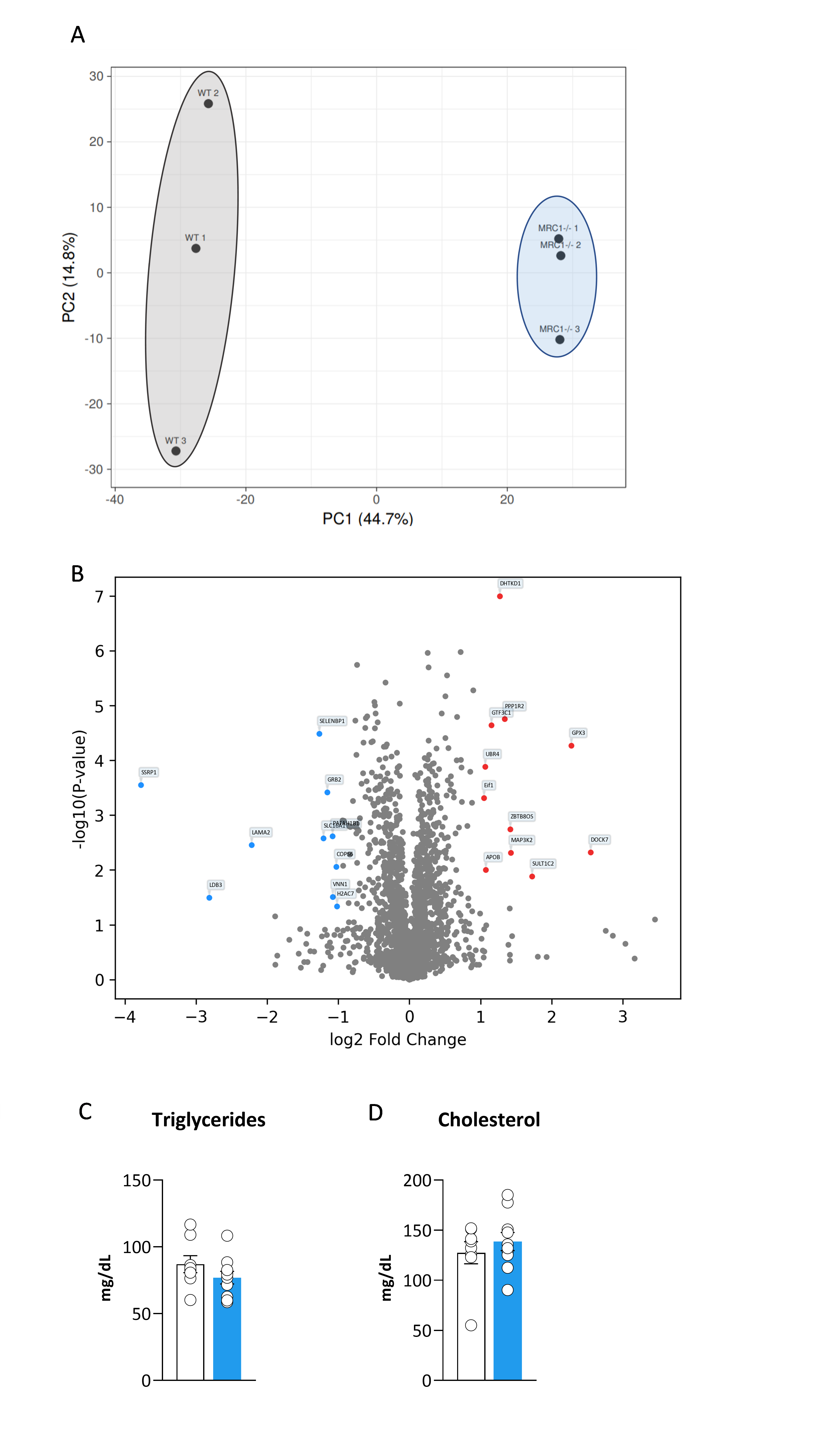

Supplement: Supplementary file 1 [file metabolites-12-01205-s001.zip › Supplementary Figure 6.png]
